# Using the Family to Combat Childhood and Adult Obesity

**Published:** 2009-06-15

**Authors:** Kenneth J. Gruber, Lauren A. Haldeman

**Affiliations:** School of Human Environmental Sciences, University of North Carolina at Greensboro; University of North Carolina at Greensboro, Greensboro, North Carolina

## Abstract

The purpose of this article is to emphasize the value of the family as a source of behavior change, particularly with respect to attaining achievable goals of weight loss and regular physical activity for youth and their families. We present a review of the literature, providing support for the value of the family in influencing children to form good diet and exercise behaviors and as a source of support and motivation for individuals seeking to lose or control their weight and to start and maintain a physically active lifestyle. Recognizing the importance of family behavior in the development of weight control and weight loss activities is essential. Future work should focus on identifying measurable parameters of family-level weight control behaviors and ways to apply those parameters to help create new interventions that use the strengths of the family for achieving weight control goals.

## Introduction

The extensiveness of the obesity issue and the potential for obesity to affect the quality of life of individuals and families underscore the urgent need for actions that can produce safe weight loss and result in effective weight management (1). The solution seems simple — take in fewer calories than you expend — but for most people this remedy is challenging. Diets and exercise routines can fail for many reasons. In part, this failure occurs because achieving weight loss through dieting or exercise requires maintenance of behavior change, which is difficult to sustain unless people have support (2-4). Support occurs most readily in a social environment that facilitates healthy eating and health-promoting exercise. Many efforts that help people to achieve weight loss fail to establish the supportive social and interpersonal context that can reinforce and help maintain weight loss–related behavior (5). Effective approaches should include these contextual influences and focus on making changes in the environment rather than in the individual. The social context most likely to support making healthy behavior changes is the family.

## Why a Family-Based Approach?

For many people, the family is a major mechanism of influence in effecting change both in other family members and in themselves (6). The concept of family has many connotations. For the purposes of this review we believe "family" should be defined inclusively rather than exclusively, similar to Medalie and Cole-Kelly's (7) description of a family as a complex of configurations representing census, biologic, household family, and functional family connections. We add the observation that family includes a parent-child connection and a sharing of responsibilities that functions for the welfare of both the individual members and the family unit.

The reciprocal nature of the adult-child relationship merits strong attention as a means of influencing health behavior of both children and adults (8). Efforts to achieve and maintain weight loss are more successful with family involvement (9). Positive eating behavior changes last longer if interventions are aimed at family rather than individuals' attitudes and habits (10).

It has been well established that physical, normative, and social characteristics of the family influence adoption and maintenance of health-promoting behavior. Family dynamics including family rules, emotional support, encouragement, reinforcement from other family members, and family member participation are important determinants of the family's health-behavior patterns (6). Viewed in this context, the family system is a major determinant of how and whether families engage in health-promoting physical activities (5).

Because most health behaviors are initiated in childhood, influencing the health behavior of individuals when they are children is reasonable and practical (10). It is well recognized that eating habits developed in childhood and adolescence may be difficult to change. Consequently, effecting behavior change when individuals are children is critical. The family shapes children's dietary intake and eating habits (11-13) and their physical activity patterns (14). Family influences also are present in the development and control of weight problems in children and adults (15-20).

The family is a highly suitable target for health promotion intervention because it provides many options and opportunities to communicate positive health behavior messages and change family member attitudes and behavior. Within the family context, meal planning, food shopping, meal preparation, eating, snacking, family recreation, and sedentary behaviors are all opportunities for intervention (16). The family provides the primary social learning environment for children and the primary setting for exposure to food choices, eating habits, and involvement in opportunities for play and other physical activity (21). Parental health behavior guides the development of health practices in children, and children can influence these same behaviors of their parents and siblings (10,22-24).

Reciprocal reinforcing relationships among family members are important for acquiring and maintaining new behaviors (25). The family is an ideal mutually reinforcing environment in which healthy behaviors can be introduced, accepted, and maintained (26). Epstein et al (19) reported findings from a series of weight loss interventions targeting adults and their children with different conditions of reinforcement of parents and the children, for the children only, or for general family participation. Results revealed that reinforcing weight loss for both the parent and the child produced the greatest weight loss over a 5-year period. The authors concluded that the relationship between parent and child weight loss can serve as a reciprocal reinforcer for changes in diet and other weight loss–related behaviors.

Family-based behavioral obesity treatment programs are among the most effective for combating pediatric obesity. Wrotniak et al (26) reported that concurrent treatment of children with their obese parents tends to result in positive change for both, though the effects tend to be greater and longer lasting for children. This may be the result of more changes to the eating and activity environment in the home or to more healthy diet and exercise role modeling of the parents.

### Family as a unit of measurement

Analyzing the family as a unit merits consideration (5,27-30). Blackwell and Reed (27) argue that a family-level analysis was more appropriate to accurately test the concepts and propositions of the power-control theory. They reasoned that because the family environment encompasses both shared and nonshared environmental influences and because of the differential effects of dyadic relationships within the family unit, analysis at the family level is appropriate when there is interest in the combination of effects of these relationships. Blackwell and Reed concluded that family-level data allowed them "to devise more methodologically appropriate measures and theoretically informative models than can be constructed with individual-level data" (p. 396). They further argued that family-level data provide control for "potential sources of 'shared environmental' characteristics" (p. 397).

Bonomi et al (28) suggested that to avoid over- and underestimations of health intervention cost effectiveness, a family-level assessment (eg, family functioning, family choices) is more appropriate. Because illness seldom affects a single individual but often affects the overall functioning of a family as a unit, determining the well-being of and costs borne by multiple family members is likely to represent a more accurate view of resource allocation. They suggest that a family well-being model, one that encompasses individuals within a family, relationships among those individuals, and the aggregation of the individuals constituting the unit, forms a good basis for addressing health at the family level. Their model is derived from systems theory, which posits that relationships between individuals and their family change over time in response to input and events that they experience alone and together (31).

### Family as the unit of health promotion intervention

Eating dinner together as a family has been associated with healthy weight and consumption of healthy foods (32-35). Gillman et al (33) found that intake patterns among children and older adolescents when eating dinner with their parents resulted in consumption of more fruits and vegetables, less fried food and soda, and less saturated and *trans* fat; lower glycemic loads; and more fiber and more micronutrients from food. Aside from the social context of the family, health similarities among family members make the family a good candidate for being the "unit" of health promotion intervention (36). In addition to the influence of genetic factors, fitness and health can be linked to the familial environment. Studies of eating habits (36,37), exercise routines (38), food and activity preferences (39), blood pressure levels (40-42), body weight (43,44), body composition and adiposity (45,46), and physical activity (47) have found that family members tend to share these characteristics.

### Families as a Support Context

Familial social support has been well demonstrated to be a key factor for promoting and sustaining health behavior change (2,48-50). Spousal support has been identified as an important factor influencing weight reduction among obese women with type 2 diabetes (18). Familial support has been reported effective in producing health-promoting behaviors among patients with cardiovascular disease (51) and for chronically ill family members achieving physical activity guidelines and practicing better dietary behaviors (52). Finally, family support consistently correlates positively with physical activity levels (49,53,54).

### Ethnic and sociocultural considerations in using families as a source for health promotion

Because of traditional values, social networks, patterns of inter- and intrafamilial support, food preferences, and recreational choices, ethnic and sociocultural factors must be considered. Food habits are deeply rooted in a family's culture, which represents both their ethnic and community identity (55). Families must contend with outside influences that affect the availability of preferred foods and with the introduction of new foods and different ways of food preparation. As a result, the change in dietary practices, at least among families with children, often occurs at the family level; most family members adopt new food choices and eating habits. This process is evident among immigrant groups as they assimilate into a new culture. As families become more acculturated, traditional foods are consumed less often.

It is widely recognized that ethnic and sociocultural influences create differences in health behaviors. For example, research has shown that Hispanics tend to be less knowledgeable about cardiovascular risk factors, prepare more of their foods by frying, and engage in less physical activity than whites (56). Members of ethnic groups respond differently to health promotion messages and interventions. Nader et al (57) found that white families reported more change in their dietary and physical activity habits than did Mexican American families after an intervention to reduce cardiovascular risk among school children. The use of an ecological perspective as a means for understanding maintenance and change in dietary practices among immigrant ethnic groups is also applicable to the family unit.

Hispanic families are strongly family-centric, which makes the influence of the family both a facilitator and a barrier for participation in physical activity. For many Hispanic wives and mothers, both the family and care of the home comes before self (58). To overcome this barrier, Hispanic immigrants feel that activities that involve the family, particularly their children, can provide them the necessary incentives and opportunities to be physically active (58). Thus, family-based interventions developed within the cultural context of the target audience (taking cultural considerations into account) may result in more effective dietary and physical activity behavior change.

## Family-Based Interventions

Dietary and exercise behaviors are well suited for family interventions because meals and recreational activities often involve the entire family. Lasting change is more likely when it involves the family unit because of the increased likelihood that family members will take action and sustain behaviors. Interventions that target the family unit also are likely to have a collective impact on the family. Cousins et al (54) compared a family-oriented intervention with a traditional (individualized) weight-loss program and an information-only control group involving obese Mexican American women. They found that, although the family-based individualized program was associated with significantly greater weight loss than the control group, the family-oriented (total family) intervention produced the greatest weight loss. The authors noted this occurred despite the fact that in the total family group other family members' (primarily the husbands') attendance was inconsistent, and changes in meal planning often were not followed because of the lack of full family participation. With more consistent family member participation, family-oriented interventions could potentially produce more behavior change.

### Family environment and childhood obesity

Although it has been argued that, for successful child obesity treatment, the primary agent of change should be the parent (16,21,59), it is clear that the family environment plays a critical role in both the development and reduction of obesity. Parental influence is a critical determinant of children's food preferences (60,61). Though the data are limited, research does suggest that some food preferences developed in early childhood persist into adulthood (62). Evidence indicates that direct involvement of at least 1 parent improves a child's weight management (15). Parental support has been reported as a determinant of children's involvement in physical activity (63-66). In addition, parental involvement has been identified as an important determinant influencing young girls to be physically active (14,67,68).

Family environment factors, such as parental feeding practices (45,69,70) and family mealtime behaviors (32,71), have been linked to overweight in children. Birch and Fisher (45) found in an assessment of parent-to-child weight status that heavy mothers tend to have heavy daughters and that daughters' weight status was affected by mothers' feeding practices. Mothers often exert influence over their daughters' dietary intake, which has been shown to negatively impact self-control over energy intake. Birch and Fisher also reported that among preschool children, efforts by mothers to use control and restrictive feeding practices produced the unintended consequence of poor self-control over food intake. Parent food purchasing and mealtime behaviors have also been correlated with poor dietary intake. Ayala et al (72) found that among Mexican families, children of parents who purchase foods seen on television or who purchase fast foods were more likely to consume more soda and dietary fat. They identified family support for healthful eating and eating regular meals together as "two modifiable targets for family-based interventions."

Golan and colleagues argue that to effectively combat child obesity, it is essential to create a family or home environment that promotes healthy family habits (16,59,73). Part of that environment involves the establishment of effective parenting behavior, which includes parents being informed about both appropriate nutrition and eating habits and adopting a physically active lifestyle that includes regular exercise. Epstein (15) reported that, in treating obese children, involving at least 1 parent as an active participant in the weight loss process improves short- and long-term weight regulation of children. He concluded that improved outcomes occur because factors in the shared family environment are targeted for change. In a 7-year follow-up, Golan and Crow (21) reported a significant mean reduction in percentage of overweight among members of the parent-focused group compared with members of the child-focused group. Robinson (17) notes that one of the keys to successful treatment of childhood obesity is improved parenting behavior relating to goal setting, reward immediacy, use of praise, appropriate modeling, and limit setting.

### The family as a solution to the obesity problem

Although the purpose of this article has been to emphasize the value of the family as a source of health behavior change, by no means are we arguing that individual-based interventions are neither effective nor often the best practice. We share the perspective of Baranowski and Nader (74) who suggest that rather than pit an individual approach and a family-oriented approach against each other, involving the entire family may be helpful in determining how to best promote behavior change among all its members. As Lindsay et al (75) write,

[p]arents play a critical role at home preventing childhood obesity, with their role changing at different stages of their child's development. By better understanding their own role in influencing their child's dietary practices, physical activity, sedentary behaviors, and ultimately weight status, parents can learn how to create a healthful nutrition environment in their home, provide opportunities for physical activity, discourage sedentary behaviors such as TV viewing, and serve as role models themselves. Obesity-related intervention programs can use parental involvement as one key to success in developing an environment that fosters healthy eating and physical activity among children and adolescents. (p. 179)

Because parents are often key to the development of a home environment that fosters healthful eating and participation in physical activity, their role is likely critical to most solutions to combating obesity. They reinforce and support healthy eating and exercise behaviors and may be best able to provide the necessary rewards to effect and maintain positive behavior change (15,75).

Many of the recommendations for addressing child and individual obesity and obesity-related factors, such as eating habits and exercise and physical activity patterns, are family-based. Suggestions include creating safe spaces to allow families to exercise or be physically active (76), increasing parental education and awareness (77,78), instructing parents to try to change children's eating and physical activity patterns (79), facilitating supportive family environments (80), and promoting positive parental support and modeling (81).

Most nonclinical interventions involving child and adolescent eating and physical activity patterns are school-based (82-86) rather than parent-based or family-based (87-90). Many school-based interventions, however, such as CATCH (Coordinated Approach to Child Health) (91), Hip-Hop to Health Jr. (92), and Students and Parents Actively Involved in Being Fit (93) include a family or parent component.

### The family as a barrier to obesity prevention

Because obesity tends to run in families, effective interventions should involve parents and other family members. However, this raises the question of how to best intervene with families. Epstein (15) and others (94-98) suggest that effective interventions for childhood obesity involve active participation by 1 or more parents. Parents need to learn how to talk with their children about exercising and eating well and how to encourage them to be more active (94). Many parents refuse to acknowledge that their children are obese (95,96). Some parents believe that actions that could help their children lose weight are ill-advised, so they refuse to support their engaging in strenuous activity or reducing their food consumption. In other cases cultural or familial factors affect parents' assessment of their children's weight and body image (97,98). As noted earlier, eating behaviors and physical activity habits must change, and if parents or children do not support such changes then weight of those at risk or already obese will likely not be well controlled (96).

In some cases it is not the intention of the family not to adopt or maintain healthy behaviors; other factors may prevent them from doing so. For example, in the case of a family member who needs to change dietary practices, family members may object or resist the introduction of new food choices (99,100). In other instances, family responsibilities such as child care responsibilities or managing the home are barriers to engaging in physical activity among parents (53). Roos et al (101) reported that the conflict between work and family life interfered with a Finnish sample of women and men in achieving recommended food habits or physical activity levels. Perceptions of environmental factors such as neighborhood safety also have been noted as barriers to physical activity (32).

## Need for a Theory of Family Behavior Change for Reducing Obesity

A further limitation to families providing the solution is that no theory involving family has been created to explain family involvement in promoting health behavior change (74). Because of the different ways (eg, modeling, support/encouragement, access to food, physical activity variety) a family may affect its members' dietary and exercise habits, it is difficult to conceive of 1 theory that accounts for family influence. As Baranowski and Nader (74) note, simply accounting for adolescent behavior and matching parental support influencing the adoption and maintenance of positive health behaviors is a major challenge. Behavior considered as positive and supportive in one parental-adolescent relationship may be perceived as controlling and confrontational in another. Soubhi et al (5) suggest that determining a family typology might be useful for focusing interventions to achieve behavior change so that the essential health-related message that is communicated is compatible with the family's structure, behavior, values, and beliefs.

To more effectively advance the notion that family (as defined by its members) be considered as a central unit for making behavior changes that support healthy eating and physical activity habits, recognizing the importance of family behavior in the development of weight control and weight loss activities is essential. A major challenge to determining family activity impact on individual members' weight management behaviors is the lack of this kind of framework with which specific activities are related to individual and family-level change. A framework by which the collection of individual-level data can be combined to form family-level aggregation of critical characteristics can combat this problem. This framework might capture who, how often, how much, to what extent, for how long, and how invested family members are as individuals and as a family unit to specific weight control actions and behaviors. A next step is to test the utility of such a framework.

Future work should build on the intricate relationships between diet and exercise and physical activity and food consumption built around the family environment. Achievable diet and physical activity goals are likely better enacted if determined by using the strengths and abilities of the family to develop and institute a plan agreed on by all family members. We hope we have described a perspective worthy of consideration by others who will build on our thesis and develop better means to convince individuals and families that a path to good health is through a lifestyle of dietary moderation and physical activity to the point of exhilaration and the desire to keep moving.
